# Peripapillary vessels density is closely related to cerebral white matter hyperintensities: An OCTA study

**DOI:** 10.1371/journal.pone.0312534

**Published:** 2024-10-31

**Authors:** Yuanyue Song, Zehua Lai, Kaiqi Ding, Yue Sun, Lili Zeng

**Affiliations:** 1 Department of Neurology and Institute of Neurology, Ruijin Hospital, Shanghai Jiao Tong University School of Medicine, Shanghai, China; 2 Department of Ophthalmology, Ruijin Hospital, Shanghai Jiao Tong University School of Medicine, Shanghai, China; IRCCS San Raffaele Scientific Research Institute, ITALY

## Abstract

**Background:**

Chronic cerebral hypoperfusion triggers the development of white matter hyperintensities (WMHs), common in cerebral small vessel disease (CSVD). However, conventional imaging techniques cannot visualize cerebral small vessels. The retina, a direct extension of the central nervous system, has an unclear correlation with WMHs. This study employs Optical coherence tomographic angiography (OCTA) to investigate vascular changes in the retina and explore its correlation with WMHs, aiming to provide a new method for assessing perfusion in early ischemic brain WMHs.

**Methods:**

Forty-nine patients with WMHs were stratified into mild and moderate/severe WMHs groups based on MRI findings, utilizing the Fazekas and Scheltens scales. OCTA assessed fundus vessel microcirculation. Logistic regression analyzed the correlation between ocular fundus microcirculation and WMH severity and location. Additionally, ROC curves evaluated the diagnostic efficacy of each fundus vascular microcirculation index in determining WMH severity.

**Results:**

After adjusting for multiple confounders, finding consistently indicated that the moderate/ severe WMHs group exhibited lower vessel density (VD) in the superior quadrant of the inner peripapillary region compared to the mild group [OR = 0.487, CI (0.255,0.929), p < 0.05]. ROC curves revealed that when combined with age, diabetes, and superior quadrant VD of the inner peripapillary region, specificity could be increased to 94.1%.

**Conclusion:**

Peripapillary vessel density correlates closely with the severity of cerebral WMHs. Early morphological changes due to chronic hypoperfusion may initiate from the inner layer of the optic disc, and OCTA could offer a novel method for evaluating blood perfusion in ischemic WMHs.

## 1. Introduction

White matter hyperintensities (WMHs), among the most common MRI manifestations of cerebral small vessel disease (CSVD), are prevalent even in asymptomatic elderly individuals. In those aged over 50 years, approximately half exhibit WMHs, a figure that rises to 95% by age 90 years [1]. The pathophysiological mechanisms of WMHs involve demyelination, axonal loss, and microinfarctions [2], heralding an increased risk of cognitive impairment, gait disturbances, stroke, and mortality [1, 3]. There is a significant reduction in white matter capillary density, occurring prior to histologically detectable changes in cerebral arterial structure [4]. Halting the progression of CSVD may delay the onset of dementia [5]. Cerebral small blood vessels, comprising small perforating arteries, arterioles, capillaries, and venules with diameters typically ranging from 40 to 200 μm, evade visualization by conventional imaging techniques due to spatial resolution constraints of computed tomography angiography and magnetic resonance angiography [6, 7]. Deep white matter hyperintensities (DWMH) and periventricular white matter hyperintensities (PWMH) exhibit distinct pathomechanisms [8], yet both lesions present with vascular fibrosis and lipoprotein disease, supporting the common ischemic vascular pathological mechanisms of WMH in the elderly [9]. Identifying methods to assess brain vessel condition and exploring the disparities between DWMH and PWMH is crucial.

The ophthalmic artery, along with its branches, constitutes the primary arterial blood supply to the eye, originating from branches of the internal carotid artery serving the brain [10]. These vessels represent the only small blood vessels in the brain directly observable, offering a unique insight into cerebral microvasculature to some extent [11]. While many studies focus on the macular system [12–16], the optic disc area receives comparatively less attention. Rich in nerves and blood vessels, the optic disc area houses the terminal arteriovenous system of the eye, the central arteriovenous system of the retina, which traverses the lamina cribrosa (LC) from the optic disc center and enters the optic network. We examine the eight quadrants of both the peripapillary and paramacular regions, as the retinal vascular system is known to be vulnerable to ischemic conditions, observing the site of earliest changes affected by chronic ischemia.

Optical coherence tomographic angiography (OCTA) represents a novel approach to detecting retinal blood flow and microvasculature by capturing motion contrast of red blood cells within vessels, without the need for intravenous dye injection [17]. In this study, we enrolled 49 patients and categorized them into mild and moderate/severe WMHs groups based on two distinct visual rating scales: the Fazekas scale and the Scheltens scale. Our objective was to assess the retinal macular area and optic nerve head (ONH) vascular system, identifying vulnerable areas in patients without dementia using OCTA.

## 2. Materials and methods

### 2.1. Participants

The author accessed the data on November 6, 2023 for research purposes. Asymptomatic patients diagnosed with WMHs at brain MRI scans performed for research purposes were enrolled in the study from the Department of Neurology, Ruijin Hospital, Shanghai Jiao Tong University School of Medicine between July 29, 2020 and May 24, 2023. The author has the right to obtain information that can identify individual participants during or after data collection. The study was approved by Ruijin Hospital Ethics Committee Shanghai Jiao Tong University School of Medicine and have been conducted according to the principles expressed in the Declaration of Helsinki. Forty-nine participants were included in this study, all of whom signed informed consent in accordance with the Declaration of Helsinki. Upon enrollment, participants underwent both head 3 T MRI and OCTA imaging. Inclusion criteria were as follows: (1) age between 50 and 80 years and (2) completion of a head MRI examination consistent with the diagnosis of "cerebral white matter hyperplasia" according to the "Chinese Expert Consensus on Cerebral Small Vessel Disease." Exclusion criteria for all participants were as follows: (1) presence of glaucoma, retinal perforation or detachment, or optic edema; (2) inability to cooperate in completing cognitive and emotional scale assessments; (3) severe liver and kidney diseases, tumor diseases, infectious diseases, or autoimmune disorders; (4) neurological diseases such as neurodegenerative disorders, multiple sclerosis, or peripheral neuropathy.

### 2.2. Assessment of white matter lesions

All participants underwent MRI examination, revealing hyperintense lesions in the white matter visible on T2-FLAIR and FLAIR sequences. WMHs were independently diagnosed by two experienced radiologists. The severity of WMHs was assessed using two scales with specific standards as follows: Fazekas scale, scoring up to 6 points. For PWMH, scoring criteria were as follows: 0 points for no lesions, 1 point for cap- or pencil-like linear thin lesions, 2 points for smooth halo-like lesions, and 3 points for irregular ventricular voiding high signal extending into deep white matter. For DWMH, scoring criteria were: 0 points for no lesions, 1 point for punctate lesions, 2 points for lesions beginning to fuse, and 3 points for large area fusion of lesions. For statistical comparisons, subjects were divided into two groups based on WMH burden: mild WMHs (scores ≤2) and moderate/severe WMHs (scores ≥3). Both PWMHs and DWMHs were scored, with scores ≤1 considered mild and scores ≥2 considered moderate/severe [18].

A modified Scheltens’ scale (total scores ranging from 0 to 30) was applied as follows: Periventricular high signals were graded from 0 to 6, with hat-shaped high intensities in the occipital and frontal lobes each scored from 0 to 2, and banded high signals in the lateral ventricle also scored from 0 to 2. Lesions were rated as 0 for no lesion, 1 for lesions ≤5 mm, and 2 for lesions 6–10 mm. Subcortical white matter was scored from 0 to 24, with WMLs in the frontal, parietal, occipital, and temporal lobes each rated up to 6. Scores were assigned as follows: 0 for no abnormality, 1 for lesions ≤3 mm and ≤5 in number, 2 for lesions ≤3 mm and >6, 3 for lesions 4–10 mm and ≤5, 4 for lesions 4–10 mm and ≥6, 5 for lesions >11 mm and >1, and 6 for fused lesions [19].

### 2.3. Optical coherence tomography angiography

**Fig 1:**
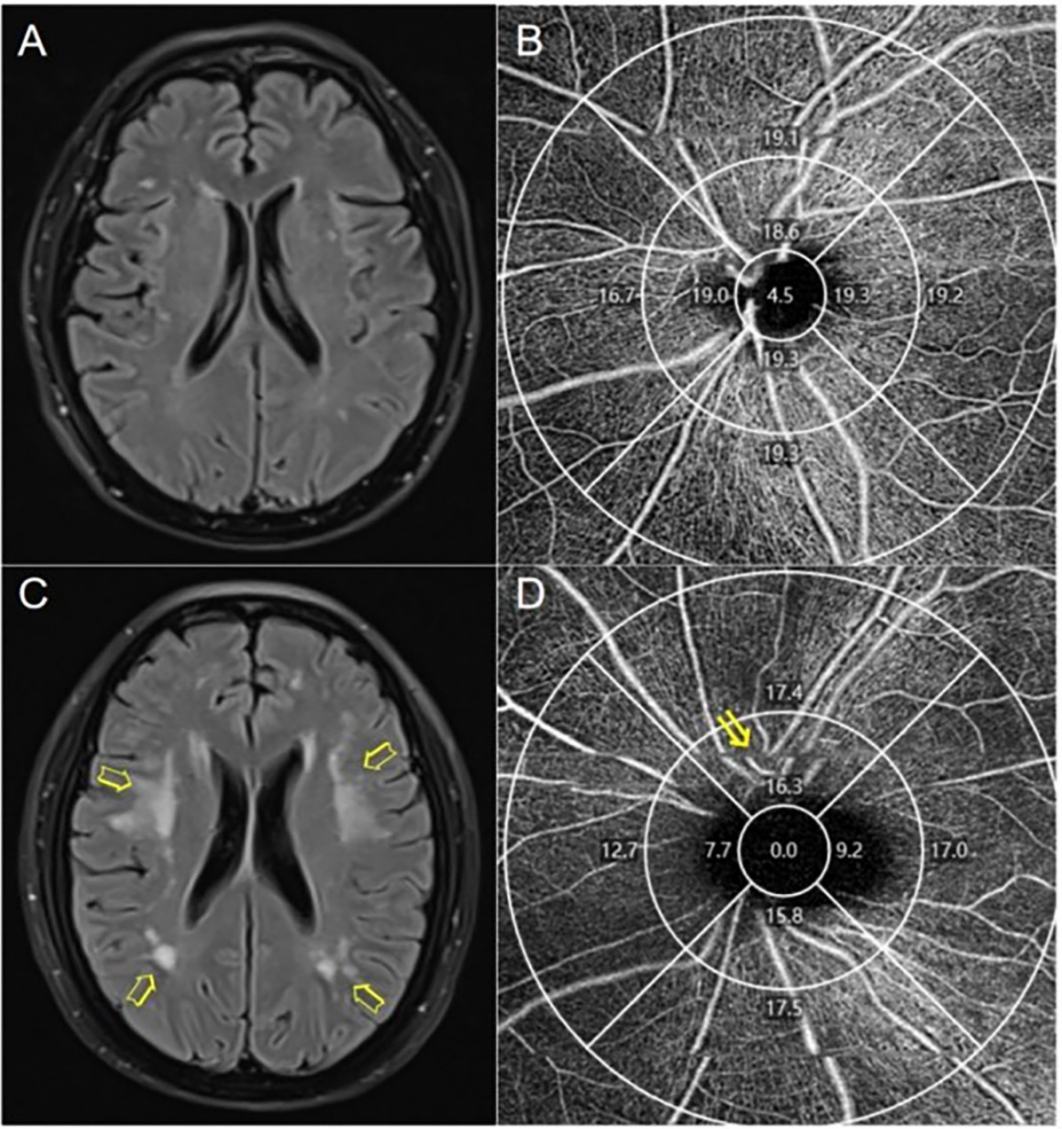
Representative images of OCT-A and brain MRI from study participants. (A) Mild white matter lesions (Fazekas scale score = 1, Scheltens scale score = 7). (B) Peripapillary vessel density (VD) of a patient with mild white matter lesions. (C) Severe white matter lesions (Fazekas scale score = 6, Scheltens scale score = 25). (D) Patient with severe WMH (arrows) showing decreased peripapillary vascular density in the superior quadrant of the inner ring (arrows).

All participants underwent OCTA (CIRRUS AngioPlex, Germany). The OCT exam was completed in approximately 5–10 minutes, with no pupil dilation required and both eyes were scanned separately. The CIRRUS AngioPlex OCTA system utilizes a wavelength of 840 nm with a scan speed of 68,000 A-scans per second. The axial resolution is 5 μm (Optical, in tissue), and the transverse resolution is 15 μm (Optical, in tissue). Each patient underwent scanning imaging with a diameter of 6 x 6 mm, from the inner limiting membrane to the inner plexiform layer, centered around the optic disc and macula. Data were analyzed using the OCT Angiography Ratio Analysis processing software. An automatic segmentation method was employed to obtain measures of the superficial vascular plexus, with quantification of vessel density (VD) expressed as the percentage of area covered by vessels. The central area (1 mm circle) was excluded from the analysis. The peripapillary and parafoveal areas, defined by two concentric rings measuring 1- and 3-mm diameter, respectively, were subdivided into four quadrants: nasal, superior, temporal, and inferior (Fig 1). Both eyes of each individual were used for the analysis.

### 2.4. Statistical analysis

Data were collected and analyzed using SPSS version 26.0 for Windows (IBM Co., Armonk, NY, USA). When both eyes of a patient were included, we used the mean interocular values for statistical analysis. Continuous variables were presented as means (standard deviations), non-normally distributed variables as medians (interquartile range—the range between the 25th and 75th percentiles), and categorical variables as frequencies (percentages). Univariate and multivariate logistic analyses were conducted to explore the relationship between OCTA parameters and WMH. For univariate analysis, Student’s t-test and X2 test were employed for continuous and categorical variables, respectively. Non-normally distributed variables were analyzed using the Mann-Whitney U-test and presented as medians (interquartile ranges, IQRs). Variables showing significant differences (p < 0.1) in the univariate analysis were included in the multivariate logistic model. All p-values were two-tailed, and significance was defined as p < 0.05.

## 3. Results

### 3.1. Demographics, clinical characteristics and OCTA parameters

Out of 56 initially screened inpatients, 49 were enrolled in the study after meeting inclusion/exclusion criteria. Exclusions included poor OCT-A image quality (n = 6) and ocular disease (n = 1). Additionally, 4 eyes were excluded due to poor image quality. Representative images of OCT-A and brain MRIs from study participants are shown in Fig 1. Using the Fazekas scale, 17 individuals were classified into the mild WMHs group, while 32 individuals were categorized into the moderate/severe WMHs group. Similarly, according to the Scheltens scale, 18 individuals were assigned to the mild WMHs group, and 31 individuals were placed in the moderate/severe WMHs group. The mean ± SD age in the mild and moderate/severe groups based on the Scheltens scale was 63.83 ± 6.47 years and 67.65 ± 6.55 years, respectively, with a tendency associated with diabetes. For the moderate/severe WMHs group, both peripapillary vessel density (VD) and perfusion density (PD) were significantly lower than those in the mild WMHs group, particularly in the inner ring (p < 0.05). This trend was also observed in the temporal and superior quadrants based on the Fazekas scale (p < 0.05), and in the temporal, superior, and inferior quadrants according to the Scheltens scale (p < 0.05) (Table 1).

**Table 1:** Comparison of clinical characteristics and OCTA parameters between different WMH groups.

	Fazekas scales		P value	Scheltens scales		P value
	≤2	>2		≤10	>10	
n	17	32		18	31	
Age (years)	64.82(6.39)	67.00(6.86)	0.285	63.83(6.47)	67.65(6.55)	**0.054**
Sex (male/female)	9/8	15/17	0.686	8/10	16/15	0.628
Hypertension (%)	7(41.2)	13(40.6)	0.97	7(38.9)	13(41.9)	0.834
Diabetes (%)	1(5.9)	8(25)	0.1	1(5.6)	8(25.8)	**0.078**
Hyperlipidemia (%)	8(47.1)	16(50)	0.845	10(55.6)	14(45.2)	0.483
Heart diseases (%)	1(5.9)	2(6.3)	0.959	1(5.6)	2(6.5)	0.9
Present smokers (%)	3(17.6)	7(21.9)	0.727	3(16.7)	7(22.6)	0.62
Present drinkers (%)	3(17.6)	4(12.5)	0.624	2(11.0)	5(16.1)	0.628
BMI	23.27(3.12)	23.19(3.14)	0.938	23.04(3.14)	23.31(3.12)	0.776
MoCA	26.00(6.25)	27.00(4.00)	0.709	26.00(5.50)	27.00(4.00)	0.755
Eyesight	1.000(0.375)	0.800(0.450)	0.229	1.000(0.350)	0.775(0.463)	0.245
IOP	13.88(2.43)	14.15(3.53)	0.479	13.85(2.63)	14.28(3.43)	0.578
Ocular axis	23.88(0.89)	23.93(1.32)	0.884	23.75(1.01)	24.00(1.27)	0.469
Vessel density						
Peripapillary						
Whole	17.55(2.00)	16.75(2.35)	0.148	17.55(2.23)	16.65(2.30)	0.051
Inner ring	18.60(3.05)	16.88(2.30)	**0.03**	18.39(2.89)	16.87(2.16)	**0.019**
Temporal	18.70(3.69)	16.50(3.75)	**0.04**	18.70(3.73)	16.38(3.70)	**0.017**
Superior	18.95(1.83)	18.00(2.80)	**0.007**	18.90(1.70)	17.95(2.85)	**0.007**
Nasal	17.23(5.28)	16.60(4.25)	0.225	17.35(4.75)	16.38(4.38)	0.241
Inferior	18.68(2.18)	17.70(2.50)	**0.058**	18.95(2.28)	17.45(2.48)	**0.028**
Outer ring	17.78(2.81)	17.20(2.99)	0.392	18.21(2.86)	17.19(3.01)	0.112
Temporal	18.63(2.69)	17.65(2.60)	0.276	18.70(2.13)	17.40(2.86)	**0.097**
Superior	18.25(1.96)	18.00(2.70)	0.375	18.35(1.95)	18.00(2.76)	0.268
Nasal	16.70(3.70)	16.30(4.35)	0.574	16.73(3.41)	16.10(4.45)	0.374
Inferior	18.55(2.83)	18.35(2.80)	0.369	19.05(2.63)	17.88(2.83)	0.129
Macula						
Whole	13.55(4.75)	13.00(5.30)	0.763	14.13(5.54)	12.73(4.16)	0.537
Inner ring	13.01(3.82)	13.25(5.89)	0.67	13.38(4.31)	13.01(5.70)	0.413
Temporal	13.40(5.64)	12.30(6.80)	0.849	14.15(4.93)	11.68(6.38)	0.203
Superior	12.65(4.21)	12.90(6.55)	0.814	12.95(5.03)	12.83(6.38)	0.394
Nasal	12.20(6.08)	14.70(7.20)	0.24	12.53(6.04)	12.63(6.79)	0.949
Inferior	12.35(2.98)	12.79(4.90)	0.741	13.20(4.53)	12.73(5.34)	0.561
Outer ring	14.79(4.41)	13.93(4.80)	0.889	15.40(4.39)	13.68(4.84)	0.393
Temporal	13.05(4.60)	12.28(7.40)	0.628	14.10(4.73)	12.05(7.63)	0.228
Superior	15.15(4.84)	14.18(4.31)	0.827	15.50(5.28)	13.65(3.43)	0.375
Nasal	16.05(3.73)	16.55(3.88)	0.608	17.08(4.44)	16.33(4.06)	0.884
Inferior	13.98(2.67)	13.70(3.73)	0.789	14.70(4.33)	13.28(5.20)	0.321
Perfusion density						
Peripapillary						
Whole	0.442(0.055)	0.420(0.067)	0.178	0.444(0.061)	0.418(0.064)	**0.044**
Inner ring	0.479(0.089)	0.443(0.068)	**0.057**	0.476(0.083)	0.439(0.064)	**0.031**
Temporal	0.449(0.077)	0.392(0.090)	**0.036**	0.442(0.081)	0.392(0.093)	**0.018**
Superior	0.500(0.059)	0.476(0.073)	**0.029**	0.501(0.058)	0.473(0.080)	**0.017**
Nasal	0.469(0.116)	0.452(0.133)	0.302	0.472(0.110)	0.451(0.134)	0.223
Inferior	0.498(0.050)	0.470(0.077)	**0.058**	0.499(0.053)	0.470(0.078)	**0.034**
Outer ring	0.450(0.073)	0.430(0.088)	0.439	0.459(0.084)	0.429(0.079)	0.109
Temporal	0.455(0.066)	0.427(0.069)	0.23	0.457(0.054)	0.423(0.074)	**0.086**
Superior	0.467(0.042)	0.473(0.080)	0.582	0.468(0.042)	0.467(0.077)	0.319
Nasal	0.425(0.102)	0.408(0.120)	0.716	0.426(0.103)	0.405(0.115)	0.289
Inferior	0.468(0.071)	0.463(0.079)	0.575	0.479(0.068)	0.451(0.078)	0.138
Macula						
Whole	0.327(0.124)	0.312(0.137)	0.738	0.344(0.148)	0.304(0.107)	0.53
Inner ring	0.302(0.095)	0.306(0.154)	0.629	0.315(0.106)	0.299(0.141)	0.425
Temporal	0.292(0.080)	0.276(0.126)	0.651	0.320(0.121)	0.268(0.168)	0.153
Superior	0.300(0.101)	0.296(0.162)	0.796	0.306(0.118)	0.295(0.151)	0.382
Nasal	0.284(0.136)	0.340(0.180)	0.203	0.289(0.148)	0.288(0.188)	0.907
Inferior	0.285(0.074)	0.300(0.119)	0.641	0.313(0.105)	0.294(0.130)	0.594
Outer ring	0.364(0.111)	0.334(0.129)	0.99	0.377(0.117)	0.328(0.132)	0.421
Temporal	0.315(0.119)	0.291(0.197)	0.695	0.340(0.117)	0.285(0.192)	0.25
Superior	0.376(0.120)	0.346(0.119)	0.991	0.385(0.131)	0.322(0.100)	0.381
Nasal	0.386(0.095)	0.389(0.110)	0.572	0.413(0.114)	0.386(0.124)	0.904
Inferior	0.339(0.071)	0.331(0.099)	0.796	0.370(0.105)	0.315(0.147)	0.295
FAZ area (mm^2^)	0.234(0.078)	0.223(0.082)	0.657	0.233(0.079)	0.215(0.096)	0.456
FAZ perimeter(mm)	2.02(0.32)	1.94(0.38)	0.495	2.02(0.37)	1.95(0.51)	0.33
FAZ circularity	0.695(0.028)	0.703(0.044)	0.486	0.703(0.021)	0.703(0.076)	0.764

BMI: Body Mass Index; MoCA: Montreal Cognitive Assessment; IOP: intra-ocular pressure; FAZ: Foveal Avascular Zone

### 3.2. Correlation and predictive efficacy of OCTA for the severity of WMHs

#### 3.2.1. The association between OCTA and the severity of WMHs

Regression analyses indicated that regional measures of peripapillary VD could distinguish between categories on visual scales (Table 2). Specifically, superior VD in the inner peripapillary region (p = 0.032) was significantly associated with the Fazekas scale categories. Furthermore, age (p = 0.031) and diabetes (p = 0.046) were positively correlated with WMHs, while superior VD in the inner peripapillary area (p = 0.029) trended negatively with the Scheltens scale. Since retinal VD can be influenced by the presence of diabetic retinopathy, we conducted a subanalysis excluding patients with diabetes to minimize this effect. In this subset, we similarly observed an association between peripapillary VD in the superior quadrant and WMHs (S1 and S2 Tables).

**Table 2:** Logistic regression analyses on related factors of patients with WMH.

	B-value	S.E value	Wald value	P-value	OR	OR 95% CI
Fazekas scales						
Superior VD of inner Peripapillary	-0.621	0.29	4.592	**0.032**	0.537	0.304–0.948
Scheltens scales						
Age	0.151	0.07	4.649	**0.031**	1.163	1.014–1.335
Diabetes	2.671	1.34	3.973	**0.046**	14.452	1.046–199.748
Superior VD of inner Peripapillary	-0.719	0.329	4.772	**0.029**	0.487	0.255–0.929

VD: vessel density

#### 3.2.2. The predictive diagnostic efficacy of retinal microcirculation for the severity of WMHs

**Fig 2:**
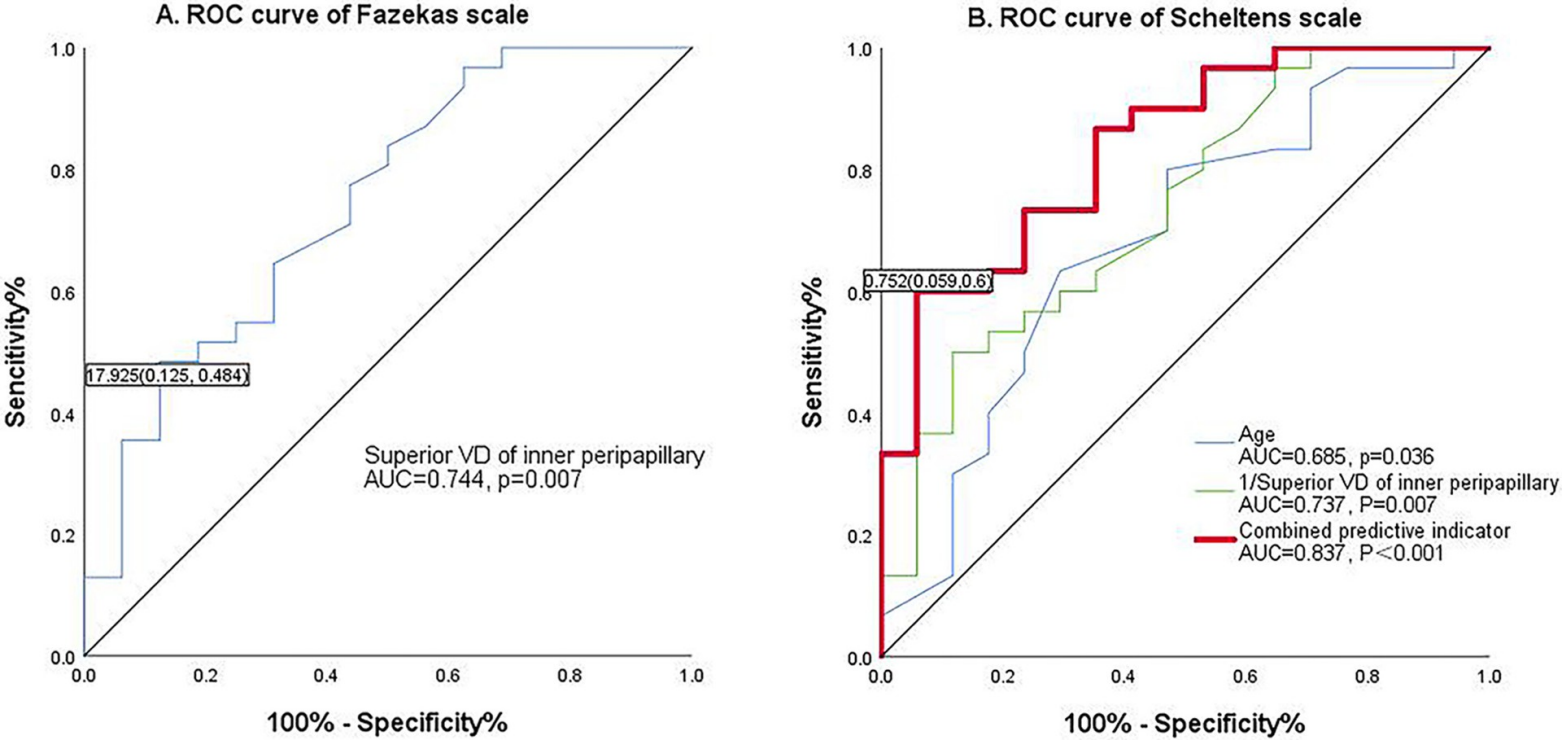
The diagnostic efficiency of WMLs. (a) ROC analysis of Fazekas scales; (b) ROC analysis of Scheltens scales.

ROC curves were used to evaluate the diagnostic abilities of clinical and demographic factors associated with OCTA parameters in patients with varying degrees of WMHs (Fig 2). Variables with p < 0.05 in the multivariable logistic regression analysis were included in the ROC curves. The ROC curves of the models for assessing WMHs severity constructed from multiple independent influences are shown in Fig 2, with an AUC of 0.744 (p = 0.007) for model 1 (using the Fazekas scale) and 0.837 (p < 0.001) for model 2 (using the Scheltens scale). Model 1, based on the Fazekas scale, demonstrated a specificity of 87.5% with a low sensitivity of 48.4%, while model 2 using the Scheltens scale showed superior specificity of 94.1% with a sensitivity of 60%.

### 3.3. Correlation and predictive efficacy of OCTA for WMHs in different brain regions

#### 3.3.1. Clinical data and OCTA parameters of DWMHs

When categorized according to the Fazekas scale, statistically significant differences were found between the groups in terms of diabetes, superior VD in the inner peripapillary region (p < 0.05). When grouped by the Scheltens scale, age, VD, and PD of the peripapillary inner ring exhibited significant differences between the two groups (p < 0.05) (Table 3).

**Table 3:** Comparison of clinical characteristics and OCTA parameters in DWMH.

	Fazekas scales		P value	Scheltens scales		P value
	≤1	>1		≤8	>8	
n	24	25		23	26	
Age (years)	65.08(5.42)	67.36(7.71)	0.239	63.57(6.12)	68.62(6.42)	**0.002**
Sex (male/female)	11/13	13/12	0.666	12/11	12/14	0.674
Hypertension (%)	9(37.5)	11(44)	0.644	8(34.8)	12(46.2)	0.419
Diabetes (%)	1(4.2)	8(32)	**0.012**	2(8.7)	7(26.9)	0.1
Hyperlipidemia (%)	12(50)	12(48)	0.889	13(56.5)	11(42.3)	0.321
Heart diseases (%)	1(4.2)	2(8)	0.576	1(4.3)	2(7.7)	0.626
Present smokers (%)	3(12.5)	7(28)	0.178	5(21.7)	5(19.2)	0.828
Present drinkers (%)	3(12.5)	4(16)	0.726	3(13.0)	4(15.4)	0.815
BMI	22.90(2.77)	23.52(3.41)	0.499	23.20(2.92)	23.23(3.30)	0.252
MoCA	27.00(5.00)	27.00(4.00)	0.486	26.50(4.25)	27.00(4.50)	0.880
Eyesight	0.800(0.400)	0.800(0.463)	0.899	1.000(0.400)	0.775(0.463)	0.597
IOP	14.40(1.85)	13.73(3.80)	0.725	13.90(3.15)	13.98(3.21)	0.569
Ocular axis	23.94(0.80)	23.87(1.48)	0.839	23.91(1.05)	23.91(1.30)	0.163
Vessel density						
Peripapillary						
Whole	17.25(1.70)	16.78(2.76)	0.45	16.53(2.16)	16.90(2.13)	0.977
Inner ring	17.59(2.55)	16.96(3.21)	0.12	18.07(2.68)	16.87(2.47)	**0.031**
Temporal	17.50(3.20)	16.38(3.69)	0.198	18.05(4.05)	16.50(3.08)	0.281
Superior	18.75(1.25)	17.85(3.19)	**0.039**	18.78(1.24)	17.80(3.05)	**0.004**
Nasal	16.65(4.00)	16.80(4.59)	0.307	17.13(3.20)	15.75(4.63)	**0.093**
Inferior	17.90(2.05)	17.70(2.80)	0.187	18.35(1.89)	17.05(2.73)	**0.082**
Outer ring	17.78(2.69)	17.26(3.33)	0.775	16.80(3.14)	17.78(2.44)	0.701
Temporal	18.65(2.70)	17.58(2.48)	0.377	18.63(3.13)	17.50(2.45)	0.549
Superior	18.35(2.35)	18.00(2.83)	0.16	18.13(2.84)	18.55(2.63)	0.235
Nasal	15.73(3.63)	16.35(4.36)	0.921	15.55(3.70)	16.30(4.23)	0.615
Inferior	18.45(3.05)	18.43(2.74)	0.807	18.50(3.08)	18.35(2.63)	0.817
Macula						
Whole	12.60(4.34)	13.43(5.10)	0.364	13.55(5.05)	12.80(5.43)	0.942
Inner ring	12.37(4.20)	13.94(5.52)	0.173	13.31(3.95)	13.23(6.32)	0.922
Temporal	12.70(6.45)	12.70(6.83)	0.489	13.40(6.31)	11.80(6.43)	0.751
Superior	12.60(3.45)	13.08(5.10)	0.322	12.88(4.35)	12.85(6.50)	0.991
Nasal	12.33(6.71)	15.18(7.29)	0.13	12.60(5.30)	12.50(9.18)	0.655
Inferior	11.96(3.48)	13.30(4.96)	0.285	12.95(4.15)	13.15(5.65)	0.462
Outer ring	13.88(4.21)	14.88(4.60)	0.375	14.79(4.71)	13.93(4.78)	0.826
Temporal	12.55(4.90)	12.70(6.95)	0.792	13.05(5.00)	12.30(6.76)	0.887
Superior	13.65(4.85)	14.90(4.30)	0.468	14.95(4.86)	14.75(4.16)	0.864
Nasal	16.00(2.68)	17.78(4.39)	0.281	16.55(2.80)	16.35(5.35)	0.902
Inferior	13.56(2.72)	14.05(3.97)	0.633	14.15(5.30)	14.10(5.80)	0.433
Perfusion density						
Peripapillary						
Whole	0.430(0.045)	0.425(0.079)	0.565	0.420(0.062)	0.430(0.061)	0.910
Inner ring	0.453(0.084)	0.448(0.084)	0.24	0.476(0.082)	0.439(0.055)	**0.046**
Temporal	0.418(0.088)	0.394(0.091)	0.25	0.428(0.096)	0.392(0.087)	0.418
Superior	0.482(0.051)	0.473(0.092)	0.114	0.501(0.054)	0.471(0.074)	**0.011**
Nasal	0.455(0.093)	0.455(0.134)	0.401	0.467(0.080)	0.444(0.147)	0.311
Inferior	0.494(0.054)	0.476(0.078)	0.317	0.497(0.045)	0.448(0.081)	**0.061**
Outer ring	0.439(0.073)	0.431(0.098)	0.912	0.413(0.080)	0.437(0.071)	0.509
Temporal	0.453(0.077)	0.426(0.069)	0.389	0.453(0.081)	0.427(0.069)	0.764
Superior	0.468(0.046)	0.467(0.088)	0.443	0.462(0.067)	0.474(0.081)	0.196
Nasal	0.405(0.103)	0.416(0.129)	0.834	0.408(0.113)	0.415(0.122)	0.668
Inferior	0.463(0.070)	0.471(0.077)	0.67	0.463(0.077)	0.465(0.071)	0.673
Macula						
Whole	0.298(0.116)	0.319(0.135)	0.265	0.327(0.129)	0.312(0.137)	0.638
Inner ring	0.279(0.107)	0.323(0.147)	0.145	0.304(0.102)	0.305(0.156)	0.882
Temporal	0.275(0.098)	0.288(0.125)	0.69	0.312(0.158)	0.278(0.165)	0.611
Superior	0.296(0.086)	0.319(0.125)	0.333	0.305(0.103)	0.295(0.168)	1
Nasal	0.284(0.155)	0.357(0.181)	**0.089**	0.291(0.114)	0.283(0.228)	0.683
Inferior	0.276(0.084)	0.314(0.121)	0.213	0.297(0.086)	0.300(0.138)	0.427
Outer ring	0.331(0.108)	0.367(0.127)	0.264	0.364(0.123)	0.333(0.127)	0.590
Temporal	0.303(0.129)	0.305(0.192)	0.717	0.315(0.139)	0.296(0.186)	0.970
Superior	0.320(0.112)	0.374(0.117)	0.297	0.376(0.123)	0.368(0.117)	0.641
Nasal	0.383(0.073)	0.425(0.151)	0.246	0.392(0.082)	0.382(0.156)	0.928
Inferior	0.326(0.073)	0.342(0.106)	0.566	0.346(0.138)	0.335(0.149)	0.591
FAZ area (mm^2^)	0.223(0.078)	0.230(0.084)	0.756	0.225(0.080)	0.215(0.135)	0.490
FAZ perimeter(mm)	1.95(0.35)	1.98(0.38)	0.771	2.00(0.28)	1.96(0.56)	0.628
FAZ circularity	0.697(0.027)	0.703(0.048)	0.582	0.700(0.020)	0.710(0.078)	0.102

BMI: Body Mass Index; MoCA: Montreal Cognitive Assessment; IOP: intra-ocular pressure; FAZ: Foveal Avascular Zone

#### 3.3.2. Clinical data and OCTA parameters of PWMHs

VD and PD in the inner layer of the optic disc were significantly reduced in cases with moderate/severe WMHs compared to those in the mild WMH group (p < 0.05) (Table 4).

**Table 4:** Comparisons of clinical characteristics and OCTA parameters in PWMH.

	Fazekas scales		P value	Scheltens scales		P value
	≤1	>1		≤2	>2	
n	18	31		19	30	
Age (years)	64.50(6.35)	67.26(6.82)	0.168	64.11(6.40)	67.60(6.66)	**0.076**
Sex (male/female)	9/9	15/16	0.913	9/10	15/15	0.858
Hypertension (%)	8(44.4)	12(38.7)	0.694	8(42.1)	12(40)	0.884
Diabetes (%)	1(5.6)	8(2.85)	**0.078**	1(5.3)	8 (26.7)	**0.059**
Hyperlipidemia (%)	9(50)	15(48.8)	0.913	10(52.6)	14(46.7)	0.684
Heart diseases (%)	1(5.6)	2(6.5)	0.9	1(5.3)	2(6.7)	0.842
Present smokers (%)	3(16.7)	7(22.6)	0.62	3(15.8)	7(23.3)	0.523
Present drinkers (%)	3(16.7)	4(12.9)	0.717	3(15.8)	4(13.3)	0.811
BMI	23.05(3.14)	23.31(3.12)	0.788	23.13(3.06)	23.27(3.17)	0.877
MoCA	26.00(5.50)	27.00(4.00)	0.755	26.50(5.50)	27.00(4.00)	0.938
Eyesight	1.000(0.350)	0.775(0.463)	0.131	1.000(0.325)	0.750(0.475)	0.141
IOP	13.90(2.63)	13.98(3.43)	0.751	13.88(2.34)	14.15(3.55)	0.627
Ocular axis	23.75(1.01)	24.01(1.27)	0.472	23.77(0.99)	24.00(1.30)	0.508
Vessel density						
Peripapillary						
Whole	17.55(2.13)	16.65(2.30)	0.068	17.58(2.19)	16.55(2.28)	**0.029**
Inner ring	18.87(2.89)	16.88(2.16)	**0.012**	18.63(2.73)	16.87(2.08)	**0.007**
Temporal	18.95(3.73)	16.38(3.70)	**0.013**	18.83(3.61)	16.25(3.48)	**0.008**
Superior	19.00(1.70)	17.95(2.83)	**0.003**	18.95(1.58)	17.90(2.85)	**0.003**
Nasal	17.45(4.85)	16.38(4.30)	0.163	17.40(4.43)	16.15(4.33)	0.12
Inferior	18.95(2.28)	17.45(2.48)	**0.022**	19.00(2.21)	17.20(2.48)	**0.011**
Outer ring	17.90(2.74)	17.20(3.01)	0.239	18.02(2.74)	17.19(3.03)	0.114
Temporal	18.65(2.58)	17.58(2.90)	0.236	18.68(2.46)	17.50(3.13)	0.139
Superior	18.35(1.95)	18.00(2.71)	0.219	18.60(1.93)	18.00(2.73)	0.168
Nasal	16.73(3.41)	16.10(4.45)	0.419	16.75(3.23)	15.90(4.18)	0.232
Inferior	18.60(2.65)	18.30(2.88)	0.278	18.83(2.48)	18.25(2.90)	0.178
Macula						
Whole	13.78(5.18)	12.90(5.29)	0.924	14.00(5.40)	12.80(4.63)	0.666
Inner ring	13.24(4.25)	13.24(5.97)	0.965	13.31(4.59)	13.23(5.68)	0.63
Temporal	13.50(5.83)	12.05(6.65)	0.514	13.83(6.03)	11.80(6.55)	0.288
Superior	12.70(4.85)	12.88(6.48)	0.833	12.83(5.19)	12.85(6.40)	0.533
Nasal	12.33(6.73)	13.73(6.99)	0.418	12.45(6.75)	12.75(6.50)	0.666
Inferior	12.67(3.18)	12.61(4.89)	0.965	12.94(3.32)	12.43(4.87)	0.689
Outer ring	14.86(4.33)	13.85(4.83)	0.615	14.93(4.22)	13.65(4.91)	0.441
Temporal	13.10(4.73)	12.05(7.63)	0.345	13.60(3.96)	11.28(8.24)	0.256
Superior	15.20(5.03)	13.65(3.98)	0.539	15.35(5.21)	13.55(3.56)	0.339
Nasal	16.45(3.24)	16.45(3.91)	0.702	16.85(4.35)	16.35(3.95)	0.911
Inferior	14.17(2.70)	13.58(3.74)	0.572	14.34(2.71)	13.44(3.74)	0.39
Perfusion density						
Peripapillary						
Whole	0.442(0.057)	0.418(0.066)	0.073	0.443(0.059)	0.416(0.064)	**0.03**
Inner ring	0.489(0.084)	0.439(0.065)	**0.025**	0.483(0.078)	0.439(0.060)	**0.017**
Temporal	0.459(0.082)	0.392(0.086)	**0.013**	0.451(0.080)	0.391(0.078)	**0.009**
Superior	0.501(0.058)	0.473(0.075)	**0.015**	0.500(0.057)	0.472(0.080)	**0.011**
Nasal	0.477(0.110)	0.452(0.131)	0.17	0.475(0.102)	0.451(0.131)	0.131
Inferior	0.499(0.053)	0.470(0.077)	**0.023**	0.500(0.049)	0.469(0.076)	**0.016**
Outer ring	0.452(0.074)	0.429(0.081)	0.244	0.454(0.080)	0.428(0.079)	0.114
Temporal	0.454(0.066)	0.426(0.075)	0.203	0.456(0.065)	0.424(0.080)	0.134
Superior	0.468(0.042)	0.467(0.077)	0.314	0.469(0.042)	0.460(0.076)	0.197
Nasal	0.427(0.094)	0.405(0.121)	0.426	0.428(0.091)	0.402(0.116)	0.211
Inferior	0.470(0.070)	0.462(0.079)	0.376	0.475(0.068)	0.461(0.079)	0.204
Macula						
Whole	0.336(0.135)	0.310(0.134)	0.898	0.344(0.144)	0.308(0.117)	0.62
Inner ring	0.303(0.105)	0.306(0.148)	0.991	0.309(0.113)	0.305(0.142)	0.67
Temporal	0.301(0.086)	0.270(0.124)	0.371	0.308(0.088)	0.265(0.123)	0.21
Superior	0.304(0.114)	0.296(0.162)	0.833	0.305(0.120)	0.296(0.156)	0.518
Nasal	0.285(0.152)	0.316(0.177)	0.4	0.285(0.161)	0.291(0.176)	0.628
Inferior	0.294(0.081)	0.295(0.118)	0.965	0.301(0.084)	0.291(0.118)	0.74
Outer ring	0.366(0.113)	0.333(0.131)	0.66	0.367(0.112)	0.328(0.133)	0.464
Temporal	0.317(0.116)	0.285(0.193)	0.393	0.329(0.097)	0.269(0.207)	0.28
Superior	0.376(0.126)	0.322(0.116)	0.649	0.381(0.129)	0.321(0.108)	0.399
Nasal	0.399(0.084)	0.386(0.122)	0.67	0.412(0.113)	0.386(0.129)	0.888
Inferior	0.345(0.074)	0.327(0.099)	0.526	0.349(0.074)	0.324(0.099)	0.36
FAZ area (mm^2^)	0.234(0.075)	0.223(0.084)	0.652	0.236(0.074)	0.221(0.085)	0.534
FAZ perimeter(mm)	2.02(0.31)	1.94(0.39)	0.486	2.03(0.31)	1.93(0.39)	0.357
FAZ circularity	0.697(0.028)	0.702(0.044)	0.642	0.696(0.028)	0.703(0.045)	0.483

BMI: Body Mass Index; MoCA: Montreal Cognitive Assessment; IOP: intra-ocular pressure; FAZ: Foveal Avascular Zone

#### 3.3.3. Association between retinal microcirculation and different WMHs sites

Multivariable logistic regression analysis showed similar results, indicating that age, diabetes, and superior VD of the inner peripapillary area were significantly associated with the severity of DWMHs and PWMHs, respectively (p < 0.05) (Table 5).

**Table 5:** Logistic regression analyses on related factors of patients with DWMHs and PWMHs.

	B-value	S.E value	Wald value	P-value	OR	OR 95% C)
DWMLs						
Fazekas scales						
Diabetes	2.581	1.155	4.994	**0.025**	13.207	1.373–127.014
Superior VD of inner peripapillary	-0.502	0.218	5.311	**0.021**	0.606	0.395–0.928
Scheltens scales						
Age	0.205	0.079	6.688	**0.01**	1.227	1.051–1.434
Superior VD of inner peripapillary	-0.563	0.26	4.678	**0.031**	0.569	0.342–0.949
PWMLs						
Fazekas scales						
Superior VD of inner peripapillary	-0.677	0.299	5.146	**0.023**	0.508	0.283–0.912
Scheltens scales						
Age	0.145	0.07	4.348	**0.037**	1.156	1.009–1.325
Diabetes	2.842	1.351	4.428	**0.035**	17.148	1.215–242.011
Superior VD of inner peripapillary	-0.794	0.344	5.338	**0.021**	0.452	0.23–0.887

DWMLs: deep white matter lesions; PWMLs: Periventricular white matter lesions; VD: vessel density

## 4. Discussion

Our study aimed to assess the link between early retinal microcirculation changes and the severity of cerebral WMHs in subclinical stages. We derived three main findings. Firstly, peripapillary VD, particularly in the superior quadrant of the inner peripapillary region, closely correlated with the severity of WMHs, encompassing subgroups of DWMH and PWMH, independent of conventional risk factors. Secondly, when age, diabetes, and superior quadrant VD of the inner peripapillary region were combined, diagnostic specificity for WMHs could be enhanced to 94.1%. Thirdly, early morphological changes attributable to chronic hypoperfusion may originate from the inner layer of the optic disc.

Cerebral small vessel disease (CSVD) constitutes a complex condition, WMHs serving as its predominant neuroimaging marker. Detecting these early physiological changes before they lead to vascular brain injury is crucial to prevent permanent damage [6, 20]. The processes of retinal vascularization during development closely mirror those of the central nervous system [11, 21]. Optical Coherence Tomography Angiography (OCTA) presents researchers with a promising approach to investigate CSVD by quantitatively analyzing retinal vascular characteristics.

Several studies have reported correlations between retinal microvascular morphology changes and cerebrovascular disease [22–32]. Two studies have demonstrated that radial peripapillary capillary network density is associated with WMHs, with some findings aligning with our observations. Additionally, they identified an association between the macula and WMHs [33, 34]. Furthermore, a recent study showed that macular microvascular signs are associated with WMH [13]. However, Ainhoa et al. suggested that macular vessel density in the superficial plexus is not indicative of cerebrovascular damage in individuals without dementia [35]. Huang et al.’s study on diabetic CSVD revealed that macular VD is associated with cerebral microbleeds and lacunar infarctions, but there is no correlation with WMHs [36]. The results of various studies are inconsistent, possibly due to differences in the severity of WMHs in the populations studied, the presence of concomitant conditions such as diabetes, associated clinical symptoms like cognitive impairment, methods used to assess WMHs, and the level of detail in OCTA assessments. Our study primarily focuses on asymptomatic patients with ischemic white matter lesions in the preclinical stage. Before enrollment, MoCA scores were assessed to eliminate interference caused by varying levels of cognitive impairment. OCTA evaluated retinal microcirculation of the macula and optic disc across multiple quadrants, aiming to identify early retinal vascular assessment indicators reflecting WMHs. This aids in the early diagnosis, evaluation, and follow-up of ischemic white matter lesions. We found that the superior quadrant of the inner layer of the optic disc correlates with the severity of WMHs, possibly indicating the earliest appearance of blood vascular change. The ONH primarily receives blood supply from the retinal circulation and the posterior ciliary artery (PCA). This blood supply pattern varies greatly among individuals and is distributed in a departmental pattern within the ONH [37]. Retinal capillary pericytes regulate pericyte tension via nitric oxide, potentially influencing capillary blood flow [38]. The terminal small arteries can automatically regulate the resistance of flow within a certain range, that is, when the perfusion pressure decreases, they expand to increase blood flow. The terminal small arteries can automatically regulate flow resistance within a certain range; when perfusion pressure decreases, they expand to increase blood flow. Therefore, in the early stages of CSVD, when blood vessel density decreases, compensatory ocular perfusion may occur, consistent with our results. This finding can better elucidate the early manifestation of retinal blood vessel changes associated with WMHs.

The biomechanical structure of the ONH may explain our results. Biomechanically, the ONH is a weak point within an otherwise strong corneo-scleral envelope [39]. The ONH and peripapillary sclera exhibit high biomechanical complexity. The ONH is nourished by the posterior ciliary artery, a branch of the ophthalmic artery that supplies the outer retinal surface [40]. These vascular systems are encased in the connective tissue of the LC, which can undergo extensive changes with age under physiological pressure and strain [39]. The mechanical properties of the LC change with age, resulting in a stiffer structure and reduced elasticity [41]. In an analysis of 5,000 finite-element eye models, it was observed that the average oxygen concentration in the inner region of the LC (30.1mmHg O_2_) was significantly lower than in the outer region (40.0 mmHg O_2_), and the average oxygen concentration in the superior (37.2 mmHg O_2_) and inferior (37.6 mmHg O_2_) regions was notably lower than in the nasal (44.2 mmHg O_2_) and temporal (43.0 mmHg O_2_) regions. Additionally, 80% of hypoxic neural tissue nodes were located in the superior and inferior regions [42]. Lee et al. found that CSVD is related to vascular density in the superior quadrant of the optic disc, which aligns with our findings [43]. Therefore, the inner-superior peripapillary area may be a potential biomarker for screening white matter lesions in the brain.

The severity of WMHs was primarily assessed using the Fazekas score, which, despite its good reliability, time-saving nature, and ease of use, was relatively simplistic [8]. Another assessment tool, the Scheltens scale, known for its reliability and validity, not only considers the size and location of lesions but also evaluates their number, showing a strong correlation with lesion volume [44]. For longitudinal observation of WMHs progression and clinical manifestations, the Scheltens scale may offer greater sensitivity and practical value. In our study, both scoring methods were utilized, revealing that the Scheltens score classification group exhibited higher diagnostic efficacy compared to the Fazekas score. By integrating age, diabetes, and vessel density in the superior quadrant of the inner peripapillary region, the AUC increased from 0.744 to 0.837, with specificity potentially rising to 94.1% when using the Scheltens scale as a reference. This suggests that the Scheltens scale is strongly recommended for studies concerning white matter lesions.

Our study has several limitations. Firstly, the relatively small sample size may limit the generalizability of our findings. Secondly, the cross-sectional design restricts our ability to infer the longitudinal effectiveness of vascular density measurements in monitoring disease progression. To address these limitations and enhance the validity of our results, future studies should prioritize longitudinal designs with larger and more diverse populations. Such research could include comparative analyses of different WMH segments and patients exhibiting cognitive impairment. Additionally, evaluating the specific location of WMHs may aid in diagnosing vascular cognitive impairment.

Our research also introduces several innovations. Firstly, we divided the optic disc and macula into more detailed regions to pinpoint areas most susceptible to damage, revealing a closer relationship between peripapillary blood vessels and WMHs. Secondly, instead of focusing solely on dementia or stroke patients, we selected preclinical patients as our study population, allowing us to detect early retinal changes and inform preventive measures. Thirdly, we utilized a scoring scale that offers a more nuanced assessment of white matter lesions, strengthening the correlation between WMHs and retinal microcirculation.

In summary, our study contributes to understanding altered microcirculation in the optic nerve and macula among WMH patients. Retinal microvascular changes serve as early, potentially more sensitive markers of WMHs before their radiological and clinical manifestations. Quantitative analysis of retinal perfusion using OCT-A emerges as a promising, non-invasive, and objective diagnostic tool for WMH. This research not only advances diagnostic capabilities but also underscores the potential of OCT-A in monitoring microcirculation and central perfusion in individuals with CSVD. We plan to follow up with patients in a future study to obtain more robust data.

## 5. Conclusion

Peripapillary vessel density closely correlates with the severity of cerebral WMHs. Early morphological changes due to chronic hypoperfusion may originate from the inner layer of the optic disc.

## Supporting information

S1 Table Comparison of clinical characteristics and OCTA parameters between different WMH groups without diabetes. (DOC)

S2 Table Logistic regression analysis of related factors in patients with WMH without diabetes. (DOC)

S1 Dataset Original data. (XLSX)
