# Supplementary material for: Peripapillary vessels density is closely related to cerebral white matter hyperintensities: An OCTA study
Source: PLoS One. 2024 Oct 31;19(10):e0312534. doi: 10.1371/journal.pone.0312534 (PMC11527194; doi:10.1371/journal.pone.0312534)
Supplement: S1 Table — (DOC) [file pone.0312534.s001.doc]

**Table 1. Comparison of clinical characteristics and OCTA parameters between different WMH groups** without diabetes

|  | Fazekas scales | | P value | Scheltens scales | | P value |
| --- | --- | --- | --- | --- | --- | --- |
|  | ≤2 ＞2 | |  | ≤10 ＞10 | |  |
| n | 16 | 24 |  | 17 | 23 |  |
| Age (years) | 64.88(6.59) | 68.17(6.13) | 0.115 | 63.82(6.67) | 69.09(5.38) | **0.009** |
| Sex (male/female) | 8/8 | 10/14 | 0.604 | 7/10 | 11/12 | 0.676 |
| Hypertension (%) | 6(37.5) | 7(29.3) | 0.581 | 6(35.3) | 7(30.4) | 0.746 |
| Hyperlipidemia (%) | 7(43.8) | 13(54.2) | 0.519 | 9(52.9) | 11(47.8) | 0.749 |
| Heart diseases (%) | 1(6.3) | 1(4.2) | 0.767 | 1(5.9) | 1(4.3) | 0.826 |
| Present smokers (%) | 2(12.5) | 4(16.7) | 0.718 | 2(11.8) | 4(17.4) | 0.622 |
| Present drinkers (%) | 3(18.8) | 1(4.2) | 0.132 | 2(11.8) | 2(8.7) | 0.749 |
| BMI | 23.10(3.15) | 22.39(2.28) | 0.421 | 22.88(3.16) | 22.52(2.26) | 0.685 |
| MoCA | 26.00(4.00) | 27.00(4.00) | 0.58 | 26.50(4.00) | 27.00(4.00) | 0.604 |
| Eyesight | 1.000(0.475) | 0.775(0.475) | 0.139 | 1.000(0.475) | 0.750(0.475) | 0.152 |
| IOP | 14.06(1.59) | 14.73(2.67) | 0.399 | 14.18(1.67) | 14.66(2.70) | 0.541 |
| Ocular axis | 23.89(0.91) | 23.64(1.14) | 0.47 | 23.75(1.04) | 23.72(1.08) | 0.939 |
| Vessel density | | | | | | |
| Peripapillary |  |  |  |  |  |  |
| Whole | 17.55(2.28) | 16.78(2.28) | 0.248 | 17.55(2.28) | 16.75(2.28) | **0.095** |
| Inner ring | 18.33(2.08) | 16.88(2.08) | **0.048** | 18.36(2.08) | 16.87(2.08) | **0.027** |
| Temporal | 17.83(2.08) | 16.14(2.65) | **0.043** | 17.93(2.06) | 16.00(2.61) | **0.018** |
| Superior | 18.90(2.85) | 17.90(2.85) | **0.013** | 18.83(2.85) | 17.80(2.85) | **0.014** |
| Nasal | 16.90(4.33) | 15.98(4.33) | 0.26 | 17.13(4.33) | 15.80(4.33) | 0.265 |
| Inferior | 18.40(2.48) | 17.15(2.48) | **0.067** | 18.68(2.48) | 17.10(2.48) | **0.033** |
| Outer ring | 17.78(3.03) | 17.56(3.03) | 0.525 | 18.41(3.03) | 17.31(3.03) | 0.143 |
| Temporal | 18.65(3.13) | 17.93(3.13) | 0.209 | 18.83(3.13) | 17.30(3.13) | **0.07** |
| Superior | 18.15(2.73) | 18.58(2.73) | 0.603 | 18.50(2.73) | 18.55(2.73) | 0.466 |
| Nasal | 16.13(4.18) | 16.30(4.18) | 0.856 | 16.70(4.18) | 16.30(4.18) | 0.601 |
| Inferior | 18.50(2.90) | 18.43(2.90) | 0.623 | 18.88(2.90) | 18.25(2.90) | 0.236 |
| Macula |  |  |  |  |  |  |
| Whole | 13.03(4.63) | 13.20(4.63) | 0.627 | 14.25(4.63) | 13.00(4.63) | 0.65 |
| Inner ring | 13.24(5.68) | 13.93(5.68) | 0.521 | 13.49(5.68) | 13.37(5.68) | 0.535 |
| Temporal | 13.50(6.55) | 12.45(6.55) | 0.988 | 14.68(6.55) | 12.05(6.55) | 0.249 |
| Superior | 12.63(2.97) | 12.32(5.04) | 0.831 | 13.29(3.20) | 11.83(4.93) | 0.306 |
| Nasal | 11.53(6.50) | 15.15(6.50) | 0.199 | 12.45(6.50) | 13.73(6.50) | 0.887 |
| Inferior | 12.28(3.06) | 12.63(5.55) | 0.798 | 13.08(3.44) | 12.03(5.43) | 0.492 |
| Outer ring | 14.86(4.91) | 14.48(4.91) | 0.903 | 15.86(4.91) | 13.85(4.91) | 0.499 |
| Temporal | 12.58(3.54) | 11.72(5.01) | 0.57 | 13.03(3.62) | 11.34(4.93) | 0.258 |
| Superior | 15.20(3.56) | 14.75(3.56) | 0.853 | 15.73(3.56) | 14.70(3.56) | 0.358 |
| Nasal | 15.70(3.95) | 16.55(3.95) | 0.581 | 17.30(3.95) | 16.23(3.95) | 0.867 |
| Inferior | 13.94(2.76) | 13.65(4.12) | 0.815 | 14.43(2.84) | 13.24(4.06) | 0.326 |
| Perfusion density | | | | | | |
| Peripapillary |  |  |  |  |  |  |
| Whole | 0.441(0.064) | 0.425(0.064) | 0.299 | 0.443(0.064) | 0.420(0.064) | **0.077** |
| Inner ring | 0.469(0.060) | 0.432(0.060) | **0.073** | 0.473(0.060) | 0.431(0.060) | **0.037** |
| Temporal | 0.428(0.051) | 0.385(0.069) | **0.045** | 0.431(0.051) | 0.382(0.068) | **0.02** |
| Superior | 0.499(0.080) | 0.471(0.080) | **0.041** | 0.496(0.080) | 0.470(0.080) | **0.023** |
| Nasal | 0.461(0.131) | 0.448(0.131) | 0.312 | 0.467(0.131) | 0.444(0.131) | 0.219 |
| Inferior | 0.496(0.076) | 0.452(0.076) | **0.055** | 0.498(0.076) | 0.448(0.076) | **0.031** |
| Outer ring | 0.447(0.079) | 0.433(0.079) | 0.575 | 0.463(0.079) | 0.428(0.079) | 0.147 |
| Temporal | 0.457(0.080) | 0.433(0.080) | 0.175 | 0.458(0.080) | 0.418(0.080) | **0.068** |
| Superior | 0.465(0.076) | 0.474(0.076) | 0.942 | 0.467(0.076) | 0.474(0.076) | 0.578 |
| Nasal | 0.416(0.116) | 0.412(0.116) | 0.988 | 0.425(0.116) | 0.408(0.116) | 0.429 |
| Inferior | 0.466(0.079) | 0.464(0.079) | 0.773 | 0.481(0.079) | 0.461(0.079) | 0.189 |
| Macula |  |  |  |  |  |  |
| Whole | 0.312(0.117) | 0.313(0.117) | 0.607 | 0.345(0.117) | 0.310(0.117) | 0.6 |
| Inner ring | 0.288(0.068) | 0.290(0.127) | 0.958 | 0.309(0.077) | 0.274(0.124) | 0.291 |
| Temporal | 0.293(0.083) | 0.278(0.141) | 0.721 | 0.315(0.091) | 0.262(0.135) | 0.181 |
| Superior | 0.292(0.071) | 0.290(0.123) | 0.946 | 0.310(0.079) | 0.277(0.120) | 0.345 |
| Nasal | 0.264(0.176) | 0.352(0.176) | 0.189 | 0.285(0.176) | 0.316(0.176) | 0.91 |
| Inferior | 0.283(0.075) | 0.296(0.135) | 0.713 | 0.305(0.087) | 0.280(0.131) | 0.501 |
| Outer ring | 0.362(0.133) | 0.351(0.133) | 0.78 | 0.386(0.133) | 0.333(0.133) | 0.521 |
| Temporal | 0.301(0.087) | 0.283(0.126) | 0.635 | 0.313(0.091) | 0.273(0.123) | 0.276 |
| Superior | 0.376(0.108) | 0.372(0.108) | 0.889 | 0.390(0.108) | 0.367(0.108) | 0.417 |
| Nasal | 0.376(0.129) | 0.389(0.129) | 0.647 | 0.412(0.129) | 0.384(0.129) | 0.769 |
| Inferior | 0.336(0.073) | 0.329(0.109) | 0.829 | 0.351(0.077) | 0.317(0.106) | 0.298 |
| FAZ area (mm^2^) | 0.231(0.079) | 0.227(0.077) | 0.873 | 0.235(0.077) | 0.223(0.079) | 0.62 |
| FAZ perimeter(mm) | 2.00(0.33) | 1.96(0.34) | 0.684 | 2.02(0.32) | 1.94(0.34) | 0.456 |
| FAZ circularity | 0.694(0.028) | 0.705(0.047) | 0.392 | 0.697(0.027) | 0.704(0.048) | 0.594 |

BMI: Body Mass Index; MoCA: Montreal Cognitive Assessment; IOP: intra-ocular pressure; FAZ: Foveal Avascular Zone
