# Supplementary material for: Peripapillary vessels density is closely related to cerebral white matter hyperintensities: An OCTA study
Source: PLoS One. 2024 Oct 31;19(10):e0312534. doi: 10.1371/journal.pone.0312534 (PMC11527194; doi:10.1371/journal.pone.0312534)
Supplement: S2 Table — (DOC) [file pone.0312534.s002.doc]

**Table 2. Logistic regression analysis of** related factors in patients with WMH without diabetes

|  | B-value | S.E value | Wald value | P-value | OR | OR 95% CI |
| --- | --- | --- | --- | --- | --- | --- |
| Fazekas scales | | | | | | |
| Superior VD of inner Peripapillary | -0.566 | 0.278 | 4.158 | **0.041** | 0.568 | 0.33-0.978 |
| Scheltens scales | | | | | | |
| Age | 0.17 | 0.078 | 4.686 | **0.03** | 1.185 | 1.016-1.382 |
| Superior VD of inner Peripapillary | -0.639 | 0.312 | 4.19 | **0.041** | 0.528 | 0.286-0.973 |
